# Activation of macrophage mediated host defense against *Salmonella typhimurium* by *Morus alba* L.

**DOI:** 10.29219/fnr.v62.1289

**Published:** 2018-02-21

**Authors:** BoYoon Chang, BongSeong Koo, HyeonCheol Lee, Joa Sub Oh, SungYeon Kim

**Affiliations:** 1 Institute of Pharmaceutical Research and Development, College of Pharmacy, Wonkwang University, Iksan, Jeonbuk, South Korea; 2 ForBioKorea Co., Ltd. Seoul, South Korea; 3 College of Pharmacy, Dankook University, Cheonan, South Korea

**Keywords:** Morus alba L, TLR4, Salmonella, immune defense, macrophage

## Abstract

**Background:**

The innate immune system plays a crucial role in the initiation and subsequent direction of adaptive immune responses, as well as in the removal of pathogens that have been targeted by an adaptive immune response.

**Objective:**

*Morus alba* L. was reported to have immunostimulatory properties that might protect against infectious diseases. However, this possibility has not yet been explored. The present study investigated the protective and immune-enhancing ability of *M. alba* L. against infectious disease and the mechanisms involved.

**Design:**

To investigate the immune-enhancing effects of *M. alba* L., we used a bacterial infection model.

**Results and discussions:**

The lifespan of mice infected with a lethal dose of *Salmonella typhimurium* (1 × 10^7^ colony forming units – CFU) was significantly extended when they were administered *M. alba* L. Furthermore, *M. alba* L. activated macrophages, monocytes, and neutrophils and induced Th1 cytokines (IL-12, IFN-γ, TNF-α) in mice infected with a sublethal dose (1 × 10^5^ CFU) of *S. typhimurium*. *M. alba* L. significantly stimulated the uptake of bacteria into peritoneal macrophages as indicated by increased phagocytosis. Peritoneal macrophages derived from C3H/HeJ mice significantly inhibited *M. alba* L. induced NO production and TNF-α secretion compared with peritoneal macrophages derived from C3H/HeN mice.

**Conclusions:**

These results suggest that the innate immune activity of *M. alba* L. against bacterial infection in mice occurs through activation of the TLR4 signaling pathway.

Bacterial infectious agents are responsible for high morbidity and mortality in humans (1). Antibiotic drugs are used to combat these pathogens; however, the rise in antibiotic-resistant pathogens has led to the development of new therapeutic agents that are effective against these bacteria (2, 3).

The immune system is our natural defense system against pathogens such as viruses, bacteria, and other agents (4). It is composed of the innate immune system and the adaptive immune system. The innate immune system is a primary defense mechanism against invading organisms, while the adaptive immune system acts as a second line of defense. Both aspects of the immune system have cellular and humoral components that mediate their protective functions. Macrophages are the most abundant cells in granulomas and have been shown to play a key role throughout the course of infection in all infected hosts, including humans and non-human primates. Once these bacteria enter a host, Toll-like receptors (TLRs) recognize a variety of microbial products, including bacterial cell wall components and endocytosed nucleic acids, thereby triggering innate immune responses (5–7). Most laboratory studies have focused on the modulation of antimicrobial peptides and innate immune mediators by microbial components (8, 9). However, the effect of herbal plant extracts on immune responses to infectious diseases is not well established. An attempt has been made to identify a potent immunostimulator from plant extracts selected for their immune pharmacological properties. Our previous study showed that *Morus alba* L. had immunostimulatory effects in macrophages (10). In addition, *M. alba* L. might have an indirect anticancer effect by enhancing immune responses through TLR4 signaling (11). However, the protective effects of *M. alba* L. against pathogens have not been examined. In the present study, we investigated the protective effect and macrophage-mediated immune responses of *M. alba* L. in mice challenged with pathogenic *Salmonella typhimurium*.

## Materials and methods

### Chemical and reagents

Roswell Park Memorial Institute medium 1640 (RPMI) and fetal bovine serum (FBS) were obtained from GIBCO (Grand Island, NY, USA). A nitric oxide (NO) detection kit was obtained from INTRON Biotechnology (Sungnam, Korea). Trizol was obtained from Invitrogen (Carlsbad, CA, USA). Interleukin (IL) 6, IL-12, interferon gamma (IFN-γ) and tumor necrosis factor alpha (TNF-α) were obtained from R&D Systems (Minneapolis, MN, USA). Penicillin, streptomycin, neutral red, 3-(4,5-dimethylthiazol-2-yl)-2,5-diphenyltetrazolium bromide, lipopolysaccharide (LPS), and all other chemicals were obtained from Sigma-Aldrich (St. Louis, MO, USA).

### Cell culture and animal care

Peritoneal macrophages were prepared from BALB/c, C3H/HeJ (wild type) and C3H/HeN (TLR4 mutant) mice as described previously (12). RAW264.7 cells were routinely cultured in Dulbecco’s Modified Eagle Medium (DMEM; Sigma-Aldrich) supplemented with 2 mM l-glutamine (Sigma-Aldrich) and 10% FBS. Briefly, peritoneal macrophages were harvested from three mice, which had been injected intraperitoneally with 3 mL of thioglycollate broth 3 days before sterile peritoneal lavage with 10 mL of Hank’s balanced salt solution. Cells were grown at 37°C in a humidified 5% CO_2_ incubator. The cells were allowed to adhere to a 96-well culture plate at 37°C in a 5% CO_2_ incubator for 3 h. Cells were grown in RPMI medium supplemented with 10% heat-inactivated FBS, 100 U/mL penicillin, and 100 μg/mL of streptomycin. *Salmonella typhimurium* was grown on Luria-Bertani (LB) agar or in LB broth, where appropriate, at 37°C under aerobic conditions. Mice were housed in specific pathogen-free (SPF) conditions at 21–24°C and between 40 and 60% relative humidity with a 12 h light-dark cycle. All animals were acclimatized for at least 1 week prior to the start of experiments. All studies were performed in accordance with the guide for animal experimentation by Wonkwang University and approved by the university’s institutional animal care and use committee (Approval No. WKU11-21).

### Preparation of standardized *M. alba* L. extract

**Fig. 1:**
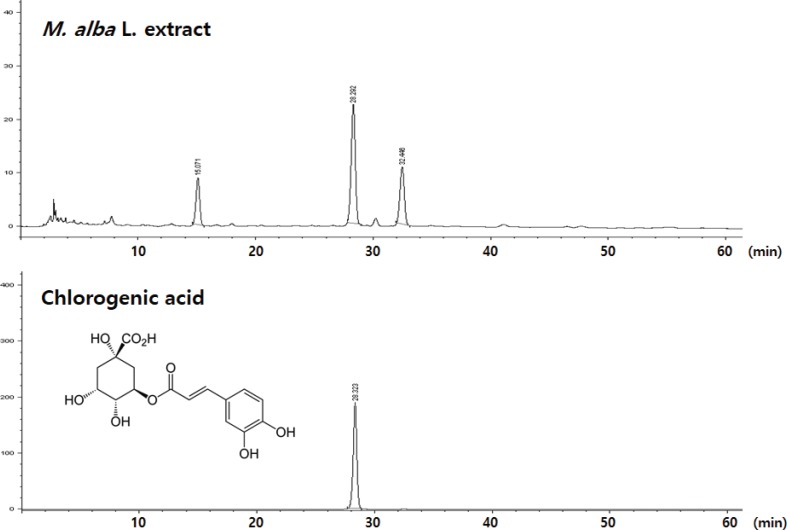
The structure and high-performance liquid chromatography (HPLC) chromatographic profile of chlorogenic acid in *M. alba* L. extract at 340 nm.

Dried fruits of *M. alba* were purchased from a local herbal market in Jeonbuk, Korea. The fruits were pulverized into powder and extracted twice with hot water (80°C) for 5 h. The solvent was removed under reduced pressure in a RV10 rotary evaporator (IKA, Staufen, Germany) to yield *M. alba* fruit hot water extract (42.8%, *w/w*). The extract was dried to a powder and kept in a closed container until use. To avoid variations in activity for different preparations, sufficient extract was obtained in one batch for use throughout the study. The content of the marker chlorogenic acid in *M. alba* L. was quantitated using high performance liquid chromatography. Results indicated that *M. alba* L. possessed 1.18 mg of chlorogenic acid per 1.0 g of extract (Fig. 1).

### Induction of *S. typhimurium* infectious model

**Fig. 2:**
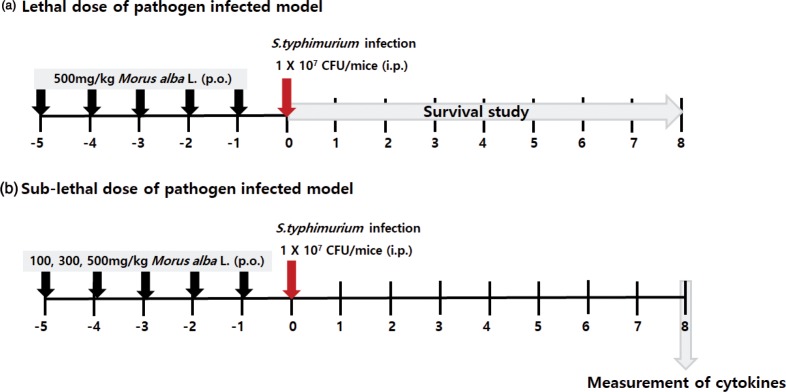
Time schedule of the experimental procedures.

SPF male ICR (Institute for Cancer Research) mice (5 weeks old and 25–27 g body weight) were purchased from Hanil Laboratory Animals (Iksan, Korea). The mice were randomly divided into two sets (Fig. 2). The first set contained three groups: a normal group (*n* = 10), *S. typhimurium-*infected group (*N* = 10), and 500 mg/kg/day *M. alba* L. + *S. typhimurium*-infected group (*n* = 10). The second set contained five groups: a normal group (*n* = 7); *S. typhimurium*-infected group (*N* = 7); 100, 300, and 500 mg/kg/day *M. alba* L. + *S. typhimurium*-infected group (*n* = 7, respectively). The normal and *S. typhimurium*-infected groups in these experiments were administered the same volume of distilled water. *M. alba* L. was dissolved in sterile double-distilled water and administered for 5 days consecutively. Twenty-four hours after the last drug administration, the mice of the first set were intraperitoneally injected with a lethal dose of bacterial suspension (10^7^ colony forming units (CFU)/mouse) to induce peritonitis. The mice in the second set were intraperitoneally injected with a sublethal dose of bacterial suspension (10^5^ CFU/mouse) to induce peritonitis. The challenged mice were fed a standard diet and water for 8 days and their survival and health were monitored twice per day throughout the experimental period. Any animal that exhibited severe clinical abnormalities, became moribund, or lost 20 % of its initial body weight was sacrificed by ethyl ether narcosis and exsanguination. At the end of the experiment, the mice were subjected to ether anesthesia. The blood samples were collected from the central vein.

### Measurement of cytokines

Blood and cell culture media were collected and then TNF-α, IL-6, IL-12, and IFN-γ production was measured by the sandwich ELISA method according to the manufacturer’s instructions (R&D Systems (TNF-α; DY410, IL-6; DY406, IL-12; DY419), Minneapolis, MN, USA and BD PharMingen (IFN-γ; RUO – 558258), CA, USA)).

### Hematology test

Blood samples were collected from the vena cava of all animals under ethyl ether anesthesia at necropsy for hematology. Blood was transferred to a container (EDTA K2; BD Biosciences, South Korea) for WBC (white blood cell), LYM (lymphocyte), MON (monocyte), and NEU (neutrophil) counts using an HORIBA (Edison, NJ, USA).

### Assay for peritoneal macrophage phagocytosis

Phagocytosis assays were performed as described by Han et al. (13). Briefly, opsonization of bacteria fluorescein-labeled *Escherichia coli* wells were opsonized by incubation for 1 h at 37°C with 5% complete mouse serum or with 5% inactivated mouse serum. After opsonization, the serum was removed by centrifugation to eliminate excess opsonins. Fluorescein-5-isothiocyanate (FITC) labeled *E. coli* cells were suspended in Hank’s balanced salt solution and added to the adherent phagocytes at a concentration of 5 × 10^5^ CFU/mL. The final ratios of bacteria to macrophages (multiplicity of infection (MOI) of 50:1) were confirmed in the final volume of 200 μL. After incubation at 37°C for 2 h, phagocytic cells were washed 3 times with phosphate-buffered saline (PBS), which was aspirated to remove non-ingested cell particles. Extracellular fluorescence was quenched by the addition of 100 μL of trypan blue. After 1 min, the FITC bacteria that had not been ingested washed away and the macrophages were rinsed twice with PBS. Next, the macrophages were lysed with lysis buffer (10 mM Tris-HCl, pH 7.5, 130 mM NaCl, 1% Triton X-100, 10 mM Na_2_HPO_4_, 10 mM Na_4_P_2_O_7_). The fluorescence intensity (relative fluorescence unit) of bacteria inside the cells was determined at excitation and emission wavelengths of 480 and 520 nm by using a spectrofluorometer (Tecan Infinite F200, Männedorf, Switzerland). The relative phagocytic activity was calculated as the percentage of fluorescence intensity in a sample supplemented with FITC bacteria compared with that with no supplementation (control).

### Intracellular killing assay

The intracellular killing assay was conducted as described by Wu et al. (14). RAW264.7 cells were seeded at a density of 5 × 10^4^ cells/well in a 96-well plate containing 200 μL complete RAW medium and incubated at 37°C for 24 h in an atmosphere of 5% CO_2._ Three colonies of each *S. typhimurium* strain were inoculated into 10 mL LB broth with appropriate antibiotics and incubated for 16 h at 37°C, with shaking at 200 rpm. Cultures were then centrifuged at 3,000 g for 10 min and resuspended in complete medium to 5 × 10^5^ CFU/mL. The culture medium was removed from the RAW264.7 cells and replaced with 200 μL of this bacterial inoculum per well, to give a MOI of 10:1. This MOI was used as the frequency of infection and was high enough to enable observation of infection events at the single-cell level in subsequent imaging experiments but minimize *Salmonella*-induced macrophage death. The cells were then incubated at 37°C for 30 min in an atmosphere of 5% CO_2_, after which the supernatant was removed from each well and 200 μL complete RAW medium containing 50 μg/mL gentamicin was added; plates were incubated for either 15 min (T0) or 2 h (T2) at 37°C for 30 min in an atmosphere of 5% CO_2_. The cells were then washed twice with 200 μL DMEM and lysed in 1% saponin, and the number of intracellular bacteria (CFU) was determined by serial dilution and plating on plate count agar. Intracellular killing percentage was calculated as: [(T0 – T2)/T0] × 100.

### NO assay

Peritoneal macrophages (1 × 10^5^ cells/well) in a 96-well plate were incubated in the presence of three concentrations of *M. alba* L. (10, 30, and 100 μg/mL) for 24 h. NO was measured by determining the concentration of its stable oxidative metabolite nitrite using a microplate assay according to a described method (10). Supernatants (100 μL) were collected and mixed with an equal volume of Griess reagent (1% sulfanilamide and 0.1% N-(1- naphthyl)ethylenediamine dihydrochloride in 5% phosphoric acid) at room temperature for 15 min. The absorbance was read at 570 nm using a microplate reader. NaNO_2_ was used as a standard.

### Statistical analysis

Data are expressed as the mean ± SD and were examined for their statistical significance of difference by analysis of variance and Student’s *t*-test. A *p*-value <0.05 was considered statistically significant.

## Results

### Effect of *M. alba* L. on pathogenic *S. typhimurium* in mice

**Fig. 3:**
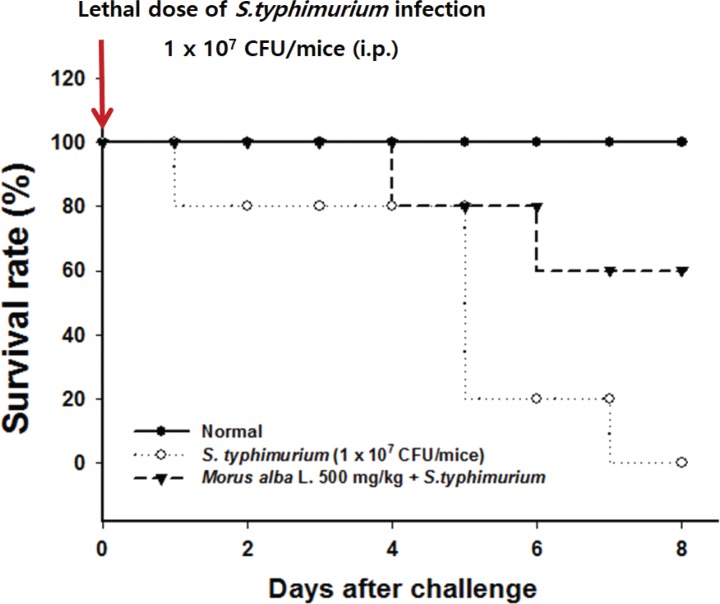
Effects of *M. alba* L. on survival rates in mice challenged with pathogenic *S. typhimurium*. *Morus alba* L. (500 mg/kg) was orally administered for 5 days to mice, which were then intraperitoneally injected with a lethal dose of a bacterial suspension (107 CFU/mouse) to induce peritonitis. The challenged mice were kept for 8 days on a standard diet with water and their survival was monitored twice a day throughout the experimental period.

To determine the therapeutic effects of *M. alba* L. on life expectancy, mice were infected with a lethal dose (1 × 10^7^ CFU) of *S. typhimurium* intraperitoneally and assessed for mortality. Figure 3 shows that the mortality rates in the normal and *S. typhimurium* groups were 100 and 0% on Day 8. By contrast, the groups treated with *M. alba* L. showed 60% had survived on Day 8. These observations demonstrate the potential of *M. alba* L. to protect mice against the lethal effects of *S. typhimurium*.

### Effects of *M. alba* L. on body and organ weight

**Fig. 4:**
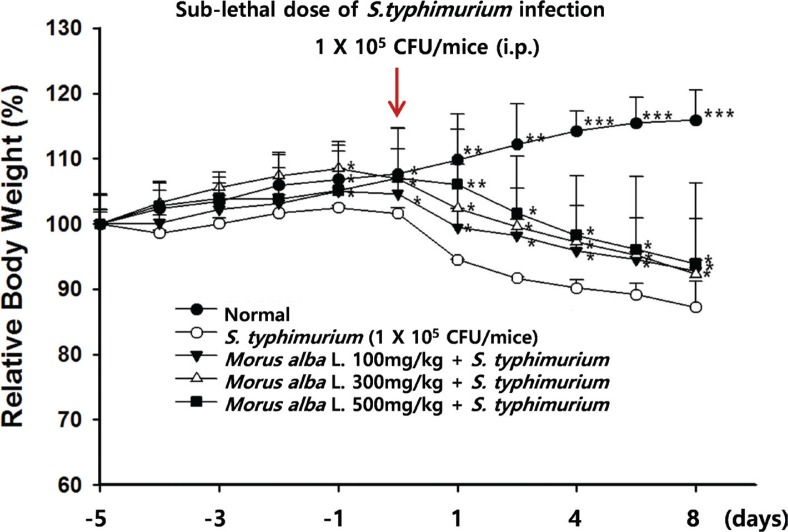
Effects of *M. alba* L. on change in body weight in mice challenged with pathogenic *S. typhimurium*. *Morus alba* L. (100, 300, or 500 mg/kg) was orally administrated for 5 days to mice, which were then intraperitoneally injected with a sublethal dose of a bacterial suspension (10^5^ CFU/mouse) to induce peritonitis. The body weight was recorded daily. The values are the means ± S.D. *p < 0.05, **p < 0.01, ***p < 0.001 compared with the *M. alba* L. group.

Body weight was monitored during the experiment to reflect the health status of mice. All the infected groups had significantly greater weight loss compared with normal mice. At Day 5, mice infected with *S. typhimurium* had lost significantly more weight compared with the *M. alba* L. group (Fig. 4). After sacrificing the animals, the organs were removed and weighed. No significant changes in the weights of livers were seen in normal, *M. alba* L. + *S. typhimurium*, or *S. typhimurium* mice (Table 1). However, spleen weights were significantly increased in animals infected with *S. typhimurium* compared with the normal group. The *M. alba* L. + *S. typhimurium* group had a significantly higher spleen weight than the *S. typhimurium* group (*p* < 0.05).

**Table 1:** Effects of *M. alba* L. on organ weight in mice challenged with pathogenic *S. typhimurium*. The values are the means ± S.D. *p < 0.05, compared with the *M. alba* L. group

Group	Dose (mg/kg)	Body weight (g)	Relative liver weight (%)	Relative Spleen weight (%)
***Normal mice***				
Saline	-	36.0 ± 1.41	5.46 ± 0.08	0.35 ± 0.08
***Mice infected with S. typhimurium (1 × 10^5^ CFU/mouse)***				
Saline		25.8 ± 1.18	6.88 ± 0.20	1.16 ± 0.08
*Morus alba* L.	100	27.6 ± 0.47	7.40 ± 0.64	1.41 ± 0.09 *
	300	26.4 ± 2.46	7.91 ± 0.11	1.55 ± 0.11 *
	500	27.0 ± 3.57	7.40 ± 0.93	1.60 ± 0.04 *

### Effects of *M. alba* L. on hematological parameters

Table 2 shows the effect of *M. alba* L. on the hematological parameters of mice. WBC count was significantly increased in the *M. alba* L. + *S. typhimurium* group (*p* < 0.05) compared with the *S. typhimurium* group. Lymphocyte count was decreased in the *M. alba* L. + *S. typhimurium* group and infected control group. Monocyte and neutrophil counts were significantly increased in the *M. alba* L. + *S. typhimurium* group and infected control group compared with the uninfected control group (*p* < 0.05 for all).

**Table 2:** Effects of *M. alba* L. on hematological parameters in mice challenged with pathogenic *S. typhimurium*. The values are the means ± S.D. *p < 0.05, compared with the *M. alba* L. group

Group	Dose (mg/kg)	WBC (10^3^/ml)	LYM (%)	MON (%)	NEU (%)
***Normal mice***					
Saline	-	4.32 ± 0.19	76.3 ± 10.28	18.4 ± 2.32	6.15 ± 3.27
***Mice infected with S. typhimurium (1 × 10^5^ CFU/mouse)***					
Saline		1.78 ± 0.24	61.3 ± 7.25	23.8 ± 1.93	16.1 ± 1.27
*Morus alba* L.	100	1.93 ± 0.27	62.7 ± 8.93	25.4 ± 1.42	13.6 ± 1.95
	300	4.22 ± 0.43 *	55.4 ± 11.75	29.5 ± 1.28 *	22.7 ± 2.95
	500	5.91 ± 1.73 *	53.4 ± 7.32	28.7 ± 1.15 *	25.1 ± 1.87 *

WBC, white blood cells; LYM, lymphocytes; MON, monocytes; NEU, neutrophils.

### Effect of *M. alba* L. on cytokine production in *S. typhimurium*-infected mice

**Fig. 5:**
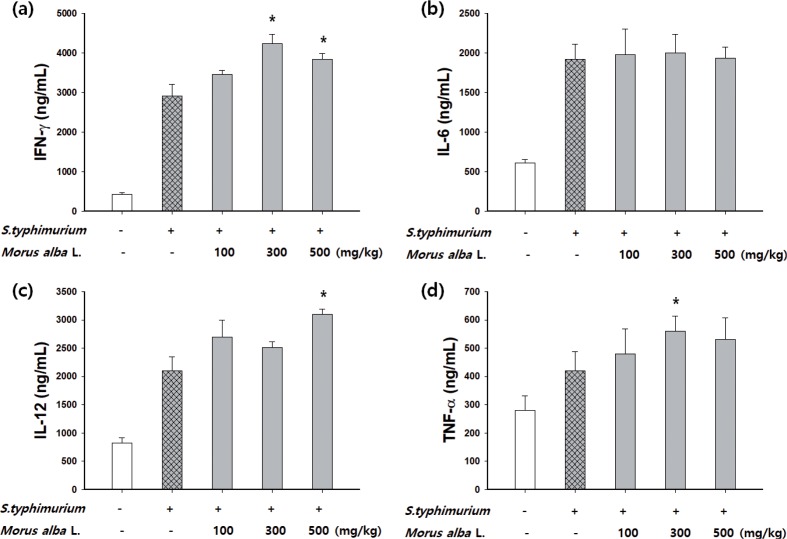
Effect of *M. alba* L. on the production of cytokines in mice challenged with pathogenic *S. typhimurium*. *Morus alba* L. (100, 300, or 500 mg/kg) was orally administrated for 5 days to mice, which were then intraperitoneally injected with a sublethal dose of a bacterial suspension (10^5^ CFU/mouse) to induce peritonitis. At the end of the experiment, the mice were subjected to ether anesthesia. Serum was collected from the blood of infected mice pre-administered with *M. alba* L. to evaluate the serum levels of (a) IFN-γ, (b) IL-6, (c) IL-12, and (d) TNF-α. The data are the means ± SD, *p < 0.05 compared with the *S. typhimurium* group.

To examine the immunomodulatory effect of *M. alba* L. on pathogenic *S. typhimurium* infection, the mice were administered *M. alba* L. (100, 300, and 500 mg/kg) for 5 days, or were not pretreated and were then challenged with 1 × 10^5^ CFU/mouse (sublethal dose) of *S. typhimurium*. After 8 days, the levels of key immune molecules, IFN-γ, TNF-α, IL-12, and IL-6 were measured in serum. The mice fed with *M. alba* L. (300 or 500 mg/kg) had higher IFN-γ, IL-12, and TNF-α production compared with the infected group (Fig. 5a, c, d). However, IL-6 production was not significantly different in the serum of mice in all groups (Fig. 5b).

### Effect of *M. alba* L. on peritoneal macrophage phagocytosis

**Fig. 6:**
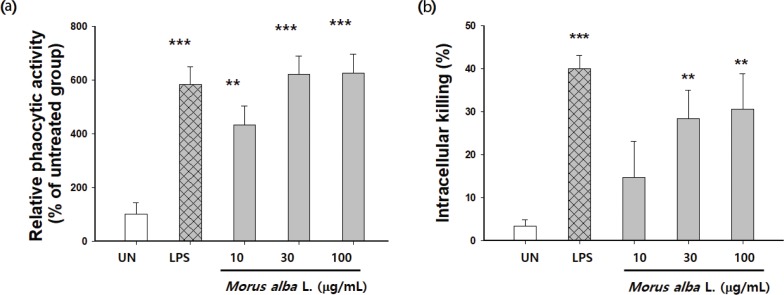
Effects of *M. alba* L. on phagocytic activity and intracellular killing. Peritoneal macrophages derived from BALB/c mice or RAW 264.7 cells were treated with 10, 30, or 100 μg/mL of *M. alba* L. for 24 h, or with LPS (1 μg/mL) as a positive control. (a) After the addition of FITC-labeled *E. coli*, the cells were incubated for 2 h. The supernatants containing unphagocytosed bacteria were removed and then lysed with Triton X-100 containing lysis buffer. Fluorescence intensities of the lysed cells were measured by using a fluorescence microplate reader. (b) After the addition of *S. typhimurium*, the cells were incubated for 2 h. Unphagocytosed bacteria were removed and the cells were lysed with 1% saponin. The number of intracellular bacteria (CFU) was determined by serial dilution and plating on plate count agar (PCA). The values are the mean ± S.D. **p < 0.01, ***p < 0.001 compared with the untreated group.

*M. alba* L. at the tested concentrations did not affect cytotoxicity (data not shown). Thus, we treated cells with *M. alba* L. at concentrations of 10, 30, and 100 μg/mL during subsequent experiments. To determine the effects of *M. alba* L. on the phagocytic activity of macrophages, the uptake of FITC-labeled *E. coli* particles was compared between *M. alba* L. treated and untreated macrophages. The phagocytosis of macrophages was increased by *M. alba* L. treatment in a dose-dependent manner (Fig. 6a). These results demonstrate that *M. alba* L. enhances macrophage phagocytosis.

### Effect of *M. alba* L. on intracellular killing

The bacteria defense functions of macrophages were further assessed by using a gentamicin protection assay, a method commonly used to test intracellular bactericidal activity with live *Salmonella* as the target. The intracellular killing of macrophages was increased by *M. alba* L. treatment in a dose-dependent manner (Fig. 6b), which demonstrated that *M. alba* L. enhanced macrophage intracellular killing.

### Effects of *M. alba* L. on peritoneal macrophages from TLR4-deficient mice

**Fig. 7:**
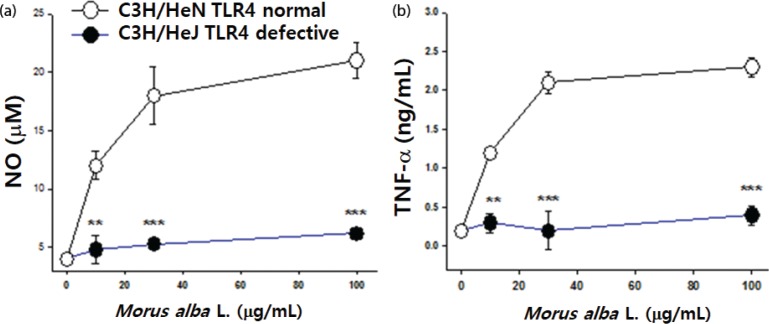
Effects of *M. alba* L. on peritoneal macrophages derived from C3H/HeN and C3H/HeJ mice. Peritoneal macrophages from C3H/HeN and C3H/HeJ mice were stimulated with *M. alba* L. or LPS (1 μg/mL) as a positive control for 24 h. (a) NO and (b) TNF-α levels were determined by ELISA. The values are the means ± S.D. **p < 0.01, ***p < 0.001, compared with C3H/HeN mice.

To investigate the involvement of TLR4 signaling in mediating the beneficial effects of *M. alba* L., we compared NO and TNF-α production in peritoneal macrophages from wild-type (C3H/HeN) or TLR4-deficient mice (C3H/HeJ). Significantly increased NO and TNF-α was detected in peritoneal macrophages from C3H/HeN but not C3H/HeJ mice after *M. alba* L. treatment (Fig. 7). Therefore, *M. alba* L. induces the production of proinflammatory cytokines via TLR4.

## Discussion

*Morus alba* L., white mulberry, is a deciduous tree that belongs to the Moraceae family, which is widely distributed in Asia. *M. alba* L. has been used worldwide in traditional medicine for the treatment of various diseases from ancient times to the present (12, 15). Previous studies have indicated that *M. alba* L. possesses various pharmacological properties, including antimicrobial (16), antioxidant (17), antitumor (18), anti-obesity (19), antihypotensive (20), neuroprotective (21, 22), and antidiabetic (23, 24) actions. In addition, our previous studies indicated that the extracts and components of *M. alba* L. fruit water extract were safe (25), immunostimulatory (10, 12, 26), and had indirect anticancer activity by enhancing immune responses mediated by TLR4 signaling (12). However, the effects and mechanisms involved in pathogen defense actions have remained elusive. Therefore, in this study, we examined the pathogen defense properties of *M. alba* L. in mice challenged with pathogenic *S. typhimurium*.

Several studies have suggested that host cells exposed to different groups of pathogens respond with common transcriptional activation programs, referred to as the *core response to infection* (27). Macrophages, monocytes, and granulocytes are involved in the non-specific defense mechanism against pathogens. The common response of macrophages to bacterial infections involves the upregulation of genes involved in M1 polarization (28).

We report that *M. alba* L. enhanced immune activity and resistance to experimental challenge with *S. typhimurium* infection. However, *M. alba* L. did not directly kill *S. typhimurium*. Therefore, the pathogen defense effect of *M. alba* L. is likely to be mediated by potentiation of the host’s defense system, such as macrophage activation, rather than by the indirect inhibition of bacterial growth.

We observed hepatomegaly and splenomegaly in *S. typhimurium* infected mice. The present results showed that the killing activity of monocytes and neutrophils was increased in *M. alba* L. treated mice infected with *S. typhimurium*, indicating its potential to enhance non-specific immune responses. Systemic *Salmonella* infection commonly induces splenomegaly in murine or human hosts. The capacity of *Salmonella* to evade killing and replicate within tissue phagocytes means that infection can quickly overwhelm local host defenses at the site of infection. Thus, inflammatory signals produced by cells in the infected tissue rapidly recruit neutrophils and inflammatory monocytes to engulf replicating bacteria. Splenomegaly can cause splenic structure change; splenic tissue structure is correlated with the recruitment and expansion of T and B cells and macrophages (29). The present study provides evidence of the induction of the macrophage phagocytic effect by *M. alba* L.

Furthermore, cytokines such as IL-12, IFN-γ, and TNF-α have a vital role in both innate and adaptive immunity and are responsible for Th1 polarization (30, 31). Because IL-12, IFN-γ, and TNF-α were upregulated in *M. alba* L. treated groups, this suggests it has important immunoregulatory activity by activating macrophages. However, Th2 cytokine production (IL-6) was unaffected. IL-12 is produced primarily by monocytes, macrophages, and other antigen-presenting cells and is essential for fighting infectious diseases and cancer. IL-12 synergizes with TNF-α and other proinflammatory cytokines to stimulate IFN-γ production, as well NK and CD8 T cell cytotoxicity (32). TNF-α is another proinflammatory cytokine and its levels in plasma are directly correlated with the ability of phagocytes to generate superoxide and the activity of iNOS and, thus, NO levels (31). One of the key requirements for the accuracy of the in vitro bactericidal assay is to eliminate the bacteria remaining extracellularly after ingestion by macrophages. For this purpose, gentamicin at a concentration of c. 50 mg/L has been widely used, as this antibiotic is believed to be incapable of penetrating the macrophage membrane. However, there are reports that such a high concentration of gentamicin penetrates HeLa cells, reaching c. 90% of the extracellular concentration after incubation for 72 h. The M1, or proinflammatory, macrophage phenotype is characterized by high levels of proinflammatory cytokines and reactive nitrogen and oxygen intermediates, the promotion of a Th1 response, and strong microbicidal and tumoricidal activity. Phagocytosis is the first step in the macrophage response to invading microorganisms and activation of phagocytosis enhances innate immune responses (33). Phagocytosis by macrophages was increased by *M. alba* L. treatment in a dose-dependent manner. The activation of intracellular killing mechanisms is critical to the antimicrobial activity of phagocytes. The pathogen internalization is followed by the fusion of phagosomes and lysosomes, which exposes the internalized pathogen to antimicrobial proteins and reactive molecules. Results indicate that *M. alba* L. is armed with such a killing capacity in a dose-dependent manner.

The first step in the modulation of cellular events is binding to receptors. TLRs play a central role in macrophage activation and the control of pathogen infections (5).

TLR4 is expressed on macrophages, dendritic cells, B cells, T cells, and endothelial cells. The role of TLR as a *M. alba* L. receptor was clearly demonstrated in our previous study (10, 12). TLR4 binds with *M. alba* L. activated signaling pathways, including MAPKs and NF-κB. Activation of MAPKs is required for the induction of NO as it controls the activation of NF-κB. In this study, the role of TLR4 as an *M. alba* L. receptor was confirmed in macrophages. To investigate this membrane receptor, we examined the effect of *M. alba* L. on primary macrophages isolated from wild-type C3H/HeN and C3H/HeJ mice that have mutant TLR4. Morus alba L. induced NO production and TNF-α in macrophages from C3H/HeN, but not from TLR4 mutated C3H/HeJ mice, which suggests that TLR4 is the membrane receptor for *M. alba* L.

In summary, our data suggest that *M. alba* L. activated macrophages via TLR4 to provide protection against *Salmonella* infection, indicating that *M. alba* L. modulates the effector functions of immunocompetent cells.

## References

[1] Qu Y, Li R, Jiang M, Wang X Sucralose increases antimicrobial resistance and stimulates recovery of Escherichia coli mutants. Curr Microbiol 2017; 74(7): 885–8. doi: 10.1007/s00284-017-1255-5. 28424940

[2] Cherazard R, Epstein M, Doan TL, Salim T, Bharti S, Smith MA Antimicrobial resistant Streptococcus pneumoniae: prevalence, mechanisms, and clinical implications. Am J Ther 2017; 24(3): e361–9. doi: 10.1097/mjt.0000000000000551. 28430673

[3] Shaker MA, Shaaban MI Formulation of carbapenems loaded gold nanoparticles to combat multi-antibiotic bacterial resistance: in vitro antibacterial study. Int J Pharm 2017; 525(1): 71–84. doi: 10.1016/j.ijpharm.2017.04.019. 28411141

[4] Luo A, Leach ST, Barres R, Hesson LB, Grimm MC, Simar D The microbiota and epigenetic regulation of T helper 17/regulatory T cells: in search of a balanced immune system. Front Immunol 2017; 8: 417. doi: 10.3389/fimmu.2017.00417. 28443096 PMC5385369

[5] McClure R, Massari P TLR-dependent human mucosal epithelial cell responses to microbial pathogens. Front Immunol 2014; 5: 386. doi: 10.3389/fimmu.2014.00386. 25161655 PMC4129373

[6] Gomes MT, Campos PC, de Almeida LA, Oliveira FS, Costa MM, Marim FM, et al The role of innate immune signals in immunity to Brucella abortus. Front Cell Infect Microbiol 2012; 2: 130. doi: 10.3389/fcimb.2012.00130. 23112959 PMC3480720

[7] Fieber C, Kovarik P Responses of innate immune cells to group A Streptococcus. Front Cell Infect Microbiol 2014; 4: 140. doi: 10.3389/fcimb.2014.00140. 25325020 PMC4183118

[8] Brito de Assis A, Dos Santos C, Dutra FP, de Oliveira Motta A, Costa FS, Navas CA, et al Assessing antibacterial potential of components of Phyllomedusa distincta skin and its associated dermal microbiota. J Chem Ecol 2016; 42(2): 139–48. doi: 10.1007/s10886-016-0665-3. 26826104

[9] Zheng Z, Wei C, Guan K, Yuan Y, Zhang Y, Ma S, et al Bacterial E3 Ubiquitin Ligase IpaH4.5 of Shigella flexneri targets TBK1 to Dampen the host antibacterial response. J Immunol 2016; 196(3): 1199–208. doi: 10.4049/jimmunol.1501045. 26700764

[10] Yang XY, Park GS, Lee MH, Chang IA, Kim YC, Kim SY, et al Toll-like receptor 4-mediated immunoregulation by the aqueous extract of Mori Fructus. Phytother Res 2009; 23(12): 1713–20. doi: 10.1002/ptr.2818. 19449343

[11] Kim SB, Chang BY, Jo YH, Lee SH, Han SB, Hwang BY, et al Macrophage activating activity of pyrrole alkaloids from Morus alba fruits. J Ethnopharmacol 2013; 145(1): 393–6. doi: 10.1016/j.jep.2012.11.007. 23164765

[12] Chang BY, Kim SB, Lee MK, Park H, Kim SY Improved chemotherapeutic activity by Morus alba fruits through immune response of toll-like receptor 4. Int J Mol Sci. 2015; 16(10): 24139–58. doi: 10.3390/ijms161024139. 26473845 PMC4632743

[13] Han EH, Choi JH, Hwang YP, Park HJ, Choi CY, Chung YC, et al Immunostimulatory activity of aqueous extract isolated from Prunella vulgaris. Food Chem Toxicol 2009; 47(1): 62–9. doi: 10.1016/j.fct.2008.10.010. 18983886

[14] Wu J, Pugh R, Laughlin RC, Andrews-Polymenis H, McClelland M, Bäumler AJ, et al High-throughput assay to phenotype *Salmonella* enterica typhimurium association, invasion, and replication in macrophages. J Vis Exp 2014; 11(90): e51759. doi: 10.3791/51759. PMC4500590 25146526

[15] Choi JW, Synytsya A, Capek P, Bleha R, Pohl R, Park YI Structural analysis and anti-obesity effect of a pectic polysaccharide isolated from Korean mulberry fruit Oddi (Morus alba L.). Carbohydr Polym 2016; 146: 187–96. doi: 10.1016/j.carbpol.2016.03.043. 27112865

[16] Tirupathi RG, Suresh BK, Ujwal KJ, Sujana P, Raoa AV, Sreedhar AS Anti-microbial principles of selected remedial plants from Southern India. Asian Pac J Trop Biomed 2011; 1(4): 298–305. doi: 10.1016/s2221-1691(11)60047-6. 23569779 PMC3614242

[17] Kujawska M, Ewertowska M, Adamska T, Ignatowicz E, Flaczyk E, Przeor M, et al Protective effect of Morus alba leaf extract on N-Nitrosodiethylamine-induced Hepatocarcinogenesis in rats. In vivo 2016; 30(6): 807–12. 27815465 10.21873/invivo.10998

[18] Yang Y, Zhang T, Xiao L, Yang L, Chen R Two new chalcones from leaves of Morus alba L. Fitoterapia. 2010; 81(6): 614–16. doi: 10.1016/j.fitote.2010.03.005. 20211228

[19] Vick SJ, Bovet D, Anderson JR How do African grey parrots (*Psittacus erithacus*) perform on a delay of gratification task? Anim Cogn 2010; 13(2): 351–8. doi: 10.1007/s10071-009-0284-2. 19777274

[20] Lee YJ, Choi DH, Kim EJ, Kim HY, Kwon TO, Kang DG, et al Hypotensive, hypolipidemic, and vascular protective effects of Morus alba L. in rats fed an atherogenic diet. Am J Chin Med 2011; 39(1): 39–52. doi: 10.1142/s0192415x11008634. 21213397

[21] Jung JW, Ko WM, Park JH, Seo KH, Oh EJ, Lee DY, et al Isoprenylated flavonoids from the root bark of Morus alba and their hepatoprotective and neuroprotective activities. Arch Pharm Res 2015; 38(11): 2066–75. doi: 10.1007/s12272-015-0613-8. 25981820

[22] Seo KH, Lee DY, Jeong RH, Lee DS, Kim YE, Hong EK, et al Neuroprotective effect of prenylated arylbenzofuran and flavonoids from morus alba fruits on glutamate-induced oxidative injury in HT22 hippocampal cells. J Med Food 2015; 18(4): 403–8. doi: 10.1089/jmf.2014.3196. 25514545

[23] Jiao Y, Wang X, Jiang X, Kong F, Wang S, Yan C Antidiabetic effects of Morus alba fruit polysaccharides on high-fat diet- and streptozotocin-induced type 2 diabetes in rats. J Ethnopharmacol 2017; 199: 119–27. doi: 10.1016/j.jep.2017. 28163112

[24] Ye M, Ke Y, Liu B, Yuan Y, Wang F, Bu S, et al Root bark of Morus alba ameliorates the depressive-like behaviors in diabetic rats. Neurosci Lett 2017; 637: 136–41. doi: 10.1016/j.neulet.2016.11.036. 27871994

[25] Chang BY, Kim SB, Lee MK, Park H, Kim SY Nonclinical safety assessment of Morus alba L. fruits: study of 90-D toxicity in Sprague Dawley Rats and genotoxicity in *Salmonella*. J Food Sci 2016; 81(5): T1328–35. doi: 10.1111/1750-3841.13285. 27075529

[26] Kim SB, Chang BY, Hwang BY, Kim SY, Lee MK Pyrrole alkaloids from the fruits of Morus alba. Bioorg Med Chem Lett 2014; 24(24): 5656–9. doi: 10.1016/j.bmcl.2014.10.073. 25467154

[27] Hurley D, McCusker MP, Fanning S, Martins M Salmonella-host interactions – modulation of the host innate immune system. Front Immunol 2014; 5: 481. doi: 10.3389/fimmu.2014.00481. 25339955 PMC4188169

[28] Zhou D, Yang K, Chen L, Zhang W, Xu Z, Zuo J, et al Promising landscape for regulating macrophage polarization: epigenetic viewpoint. Oncotarget. 2017; 8(34): 57693–706. doi: 10.18632/oncotarget.17027. 28915705 PMC5593677

[29] Chu HB, Zhang TG, Zhao JH, Jian FG, Xu YB, Wang T, Wang M, Tang JY, Sun HJ, Li K, Guo WJ, Zhu XJ Assessment of immune cells and function of the residual spleen after subtotal splenectomy due to splenomegaly in cirrhotic patients. BMC immunology 2014; 15: 42. doi: 25293512 10.1186/s12865-014-0042-3 PMC4193139

[30] Kalupahana RS, Mastroeni P, Maskell D, Blacklaws BA Activation of murine dendritic cells and macrophages induced by *Salmonella* enterica serovar Typhimurium. Immunology 2005; 115(4): 462–72. doi: 10.1111/j.1365-2567.2005.02180.x. 16011515 PMC1782185

[31] Perkins DJ, Rajaiah R, Tennant SM, Ramachandran G, Higginson EE, Dyson TN, et al Salmonella typhimurium co-opts the host type I IFN system to restrict macrophage innate immune transcriptional responses selectively. J Immunol. 2015; 195(5): 2461–71. doi: 10.4049/jimmunol.1500105. 26202980 PMC4546913

[32] Fernandez-Cabezudo MJ, Mechkarska M, Azimullah S, al-Ramadi BK Modulation of macrophage proinflammatory functions by cytokine-expressing *Salmonella* vectors. Clin Immunol 2009; 130(1): 51–60. doi: 10.1016/j.clim.2008.08.017. 18835224

[33] Hamidzadeh K, Mosser DM Purinergic signaling to terminate TLR responses in macrophages. Front Immunol 2016; 7: 74. doi: 10.3389/fimmu.2016.00074. 26973651 PMC4773587

